# Use of Natural Polymers for the Encapsulation of Eugenol by Spray Drying

**DOI:** 10.3390/pharmaceutics16101251

**Published:** 2024-09-26

**Authors:** Aitor Caballero-Román, Anna Nardi-Ricart, Roser Vila, Salvador Cañigueral, Josep R. Ticó, Montserrat Miñarro

**Affiliations:** 1 Departament de Farmàcia i Tecnologia Farmacèutica, i Fisicoquímica, Facultat de Farmàcia i Ciències de l’Alimentació, Universitat de Barcelona (UB), Avinguda Joan XXIII, 27-31, 08028 Barcelona, Spain; annanardi@ub.edu (A.N.-R.); minarromontse@ub.edu (M.M.); 2 Unitat de Farmacologia, Farmacognòsia i Terapèutica, Facultat de Farmàcia i Ciències de l’Alimentació, Universitat de Barcelona (UB), Avinguda Joan XXIII, 27-31, 08028 Barcelona, Spain; r.vila@ub.edu (R.V.); s.canigueral@ub.edu (S.C.); 3 Facultat de Farmàcia i Ciències de l’Alimentació, Universitat de Barcelona (UB), Avinguda Joan XXIII, 27-31, 08028 Barcelona, Spain; jrtico@ub.edu

**Keywords:** spray drying, eugenol, encapsulation, polymers

## Abstract

**Background:** Eugenol is a colourless or yellowish compound whose presence in clove essential oil surpasses the 75% of its composition. This phenylpropanoid, widely used as an antiseptic, anaesthetic and antioxidant, can be extracted by steam distillation from the dried flower buds of *Syzygium aromaticum* (L.). Due to its chemical instability in presence of light and air, it should be protected when developing a formulation to avoid or minimise its degradation. **Methods:** A promising approach would be encapsulation by spray drying, using natural coating products such as maltodextrin, gum arabic, and soy lecithin. To do so, a factorial design was carried out to evaluate the effect of five variables at two levels (inlet temperature, aspirator and flow rate, method of homogenisation of the emulsion and its eugenol:polymers ratio). Studied outcomes were yield and outlet temperature of the spray drying process, eugenol encapsulation efficiency, and particle size expressed as d_(0.9)_. **Results:** The best three formulations were prepared by using a lower amount of eugenol than polymers (1:2 ratio), homogenised by Ultra-Turrax^®^, and pumped to the spray dryer at 35 m^3^/h. Inlet temperature and flow rate varied in the top three formulations, but their values in the best formulation (DF22) were 130 °C and 4.5 mL/min. These microcapsules encapsulated between 47.37% and 65.69% of eugenol and were spray-dried achieving more than a 57.20% of product recovery. Their size, ranged from 22.40 μm to 55.60 μm. **Conclusions:** Overall, the whole spray drying process was optimised, and biodegradable stable polymeric microcapsules containing eugenol were successfully prepared.

## 1. Introduction

Essential oils (EOs) are volatile, odorous, and usually complex products whose origin is a secondary metabolism of certain plants [1]. They are obtained from botanically defined herbal drugs by processes as dry or steam distillation or by any other appropriate mechanical process without heat involved [2]. Traditionally, EOs have been used as natural preservatives in alimentary, cosmetic, and pharmaceutic industry [3].

Clove essential oil (CEO) is a clear yellow liquid obtained by steam distillation of the dried flower buds of *Syzygium aromaticum* (L.), an aromatic plant from the Myrtaceae family that can be found in different Asian countries and Madagascar [4,5]. CEO, a Generally Recognised As Safe (GRAS) substance by the U.S. Food and Drug Administration (FDA) [6], is mainly known for its antimicrobial and antifungal activity [7]. CEO contains eugenyl acetate, β-caryophyllene, and α-humulene, but the compound whose presence surpasses 75% of the composition of CEO is eugenol (EUG), a phenylpropanoid with several biological activities [4]. This colourless or yellowish compound, also present in other aromatic plants, such as ginger (*Zingiber officinale*), black pepper (*Piper nigrum*), or oregano (*Origanum vulgaris*), is practically insoluble in water and sensitive to air and light [4,5,8]. EUG has been widely used as an antiseptic but also possesses anaesthetic and antioxidant properties [4]. Recent studies also proved its value as an enhancer of anti-proliferative effects in resistant melanoma and other cancers, such as breast or colon, when used in combination with other drugs [4,9].

Due to the volatility of EUG and its chemical instability in the presence of light and oxygen [8], it is recommended to minimise the degradation of EUG when developing a formulation. Several techniques, such as extrusion, fluidized bed, freeze drying, or complexations, can be used [10,11]. Nonetheless, microencapsulation via spray drying could be one of the most promising approaches. This technique, widely used for the encapsulation of bioactive components, consists of the dispersion of a sample by using a pressure nozzle in a vacuum chamber with hot air flow to evaporate the moisture of the product and collect it in a glass container [12]. Some benefits of spray drying are its flexibility, high speed, economic cost, and easiness of working with different encapsulating agents or phytochemicals. Additionally, it is easy to scale at the industrial level, and it can be used in continuous operation to produce good quality particles, two relevant features in alimentary and pharmaceutical industry [11,13,14,15].

Then, encapsulation can be defined as a process where a thin coating layer is formed around living cells, solid, liquid, or gaseous particles [16]. By creating this coating layer, it is possible to improve the flow properties of powders; mask unpleasant colours, flavours, and aromas; or stabilise substances that are sensitive to light and/or oxygen. Moreover, physicochemical properties of different molecules can be improved, such as their solubility, bioavailability, or release profile. One way to encapsulate substances via spray drying, especially effective for lipophilic compounds, is the emulsion method [11,17].

To encapsulate compounds by using this method, wall materials must be chosen in order to ensure stability of the formulations. When an emulsion is prepared with an aqueous wall material and a lipophilic molecule of interest, a coating is instantly formed around the active compound when the liquid is pumped into the spray dryer. By doing so, the core would be isolated from the environment [17].

Consequently, it is mandatory that the wall materials are water-soluble and have film-forming properties to properly isolate the encapsulated product and, obviously, be biocompatible and biodegradable [3,10]. As additional features, it is better when they are GRAS substances, economic, non-immunogenic, and neutral in terms of flavour and aroma [15].

In this research paper, the discontinuous phase of the emulsion contained EUG, whereas the continuous phase of the emulsion was composed by maltodextrin (MD) and gum arabic (GA). Both polymers fulfil the previously stated requirements. Moreover, both possess film-forming capacities and good water solubility, producing low viscous solutions even at high concentrations [14,17,18,19].

MD is a cheap and widely used starch hydrolysate that is highly soluble in cold water. It is able to provide a strong barrier against degradation and oxidation of bioactive compounds by its film-forming capacities [3,15,18]. MD can be classified according to its dextrose equivalent (DE), indicative of its hydrolysis degree when compared to the starch molecule. To be an optimum wall material, DE should be between 10 and 20 [14,18].

GA is also one of the most used wall materials because of its multiple properties. This gum, obtained from acacia tree, is composed by monosaccharides, such as galactose, rhamnose, and arabinose, in combination with glucuronic acid and almost 2% of protein [14]. GA is a good emulsifier, thickening agent, and humectant [3,14], although one of its most interesting properties when encapsulating oils by spray drying is the protection that confers to volatile molecules in a wide pH range [3,15].

Nevertheless, GA is not able to protect substances from oxidation by itself. For this reason, it is normally used in combination with other polymers, such as MD, to protect the core material of the microcapsules from external agents and even high temperatures [3,14].

Besides MD and GA, soy lecithin (SL) was also added to the emulsion. This low molecular-weight compound, mainly composed of phospholipids, acted as an emulsifier and stabiliser [20]. Moreover, SL is able to decrease the oxidation of the encapsulated product and improve its encapsulation efficiency thanks to the extra stability that provides to the formulation for its amphiphilic nature [14]. Therefore, all three substances, MD, GA, and LS, act synergically when encapsulating products, such as EUG, due to their complementary properties. All used polymers in this research paper, as well as EUG from CEO, are GRAS-certified by the FDA [6,21,22,23].

Choosing good coating polymers is important; however, the spray drying conditions should be optimised as well. The most relevant process variables are inlet temperature, aspirator rate, flow rate, and ratio of polymers in relation to the active ingredient.

Therefore, the aim of this research was the optimisation of a spray drying process by means of a 2^5^ factorial design, leading to the manufacture of 32 formulations, to produce EUG microcapsules by using natural and biocompatible products as coating agents.

## 2. Materials and Methods

### 2.1. Materials

EUG 99% purity was donated by Lidervet (Tarragona, Spain). MD (C-Dry™ MD, dextrose equivalent 12–16), and SL (Emultop™ IP) were gifted by Cargill (Barcelona, Spain). GA from acacia tree, spray dried, was purchased from Merck (Darmstadt, Germany). Distilled water and Milli-Q^®^ water (Merck Millipore Milli-Q^®^ Advantage A10) were, respectively, used to prepare the polymeric solutions and to dissolve the microparticles in one assay. Hexane, as an alkanes mixture for analysis and 2-propanol pharma grade, were obtained from Panreac AppliChem (ITW Reagents; Barcelona, Spain). Azulene 99% was acquired from Alfa Aesar (Haverhill, MA, USA).

### 2.2. Experimental Design

A randomised factorial design was prepared by means of the software Statgraphics^®^ Centurion 18 (The Plains, VA, USA) to study the effect of 5 factors elevated at 2 levels by performing 32 experiments. The chosen factors, relevant in the preparation of the emulsions of the spray drying process, are presented in Table 1.

Data from all 32 assays is shown in Table A1 from Appendix A.

To study the effect of all the above-mentioned factors, four outcomes were analysed. Outcomes are listed in Table 2 by order of importance.

Moreover, additional outcomes were studied to achieve a greater characterisation of the microparticles. These parameters, not prioritised to improve EUG microcapsules, were polydispersity index (PDI), surface-weighted mean diameter (d_[3,2]_), and volume-weighted mean diameter (d_[4,3]_).

### 2.3. Emulsion Preparation

MD and GA were hydrated in distilled water for 24 h at room temperature (24 °C). Afterward, SL was added (0.5% *w*/*w*) to a 3:1 (MD:GA) mixture of the polymers. The emulsifying process and the EUG ratio in the emulsion depended on their value in the randomised factorial design. Formulations were prepared by using a high shear homogeniser (Ultra-Turrax^®^ T-25; IKA^®^-Werke, Staufen, Germany) at 15,000 rpm for 5 min or by means of a paddle stirrer (RW 20 Digital; IKA^®^-Werke, Staufen, Germany) at 390 rpm for 10 min. A certain volume of EUG was added dropwise while homogenising in all formulations, according to the design of the experiment.

### 2.4. Emulsion Characterisation

The optic microscope Leica DM 1000 LED, equipped with a Leica EC3 camera (Leica; Wetzlar, Germany), was used to characterise the emulsions. To analyse the images, the software Leica LAS EZ 3.4 was employed.

### 2.5. Spray Drying Process

**Figure 1:**
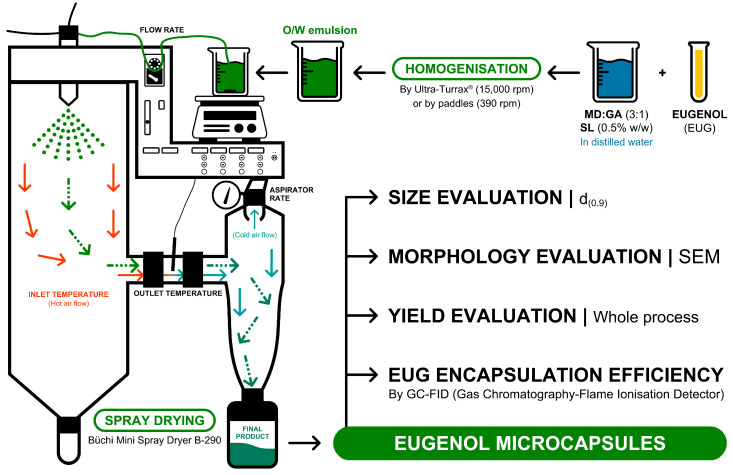
Summary of the spray drying process and study of the microcapsules. Orange arrows indicate hot air flow, controlled by inlet temperature. Cold air flow, controlled by the aspirator rate, is represented by blue arrows. Green arrows indicate the route the microcapsules follow to the collector. Icons retrieved from The Noun Project (CC BY 3.0); credits to Jonathan Li for the stirrer, ic2icon for the beaker, and Evgenii Likhachov for the arrows.

Emulsions were pumped at 1.5 mL/min or 4.5 mL/min in a Büchi Mini Spray Dryer B-290 (Büchi Ibérica, Barcelona, Spain). The design of the experiment also specified the inlet temperature (110 °C or 130 °C) and the flow rate of the drying air (28 m^3^/h or 35 m^3^/h), known as the aspirator rate. The nozzle cleaner was always set at 5. Formulations were stored in glass opaque containers at room temperature (24 °C). A summary of the technique and some of the evaluated parameters from the microcapsules are shown in Figure 1.

### 2.6. Outlet Temperature

It was registered by using the outlet temperature probe from the spray dryer when its value was stable.

### 2.7. Product Recovery

Product recovery was evaluated by calculating the yield of the spray drying process by means of Equation (1), relating the obtained EUG microcapsules and the initial polymer and EUG amount.

(1) $$\mathrm{Yield}\;(\%)=\frac{\mathrm{Final}\;\mathrm{product}}{\mathrm{MD}+\mathrm{GA}+\mathrm{SL}+\mathrm{EUG}}\times 100$$

where Final product corresponded to the obtained amount in grams of the EUG microcapsules after the spray drying process. MD, GA, SL, and EUG were, respectively, the amount in grams of MD, GA, SL, and EUG.

### 2.8. Particle Size Distribution

Particle size distribution was measured by means of a laser diffraction equipment (Mastersizer Hydro 2000 SM) equipped with a Malvern Dispersion Unit Controller (both from Malvern Instruments; Malvern, UK).

Approximately 1 g of the obtained product was suspended by means of a stirrer in 2-propanol (refractive index $n_{20}^{D}$ 1.390) to perform the assay by triplicate.

As the main parameter to evaluate particle size distribution, 90% cumulative equivalent volume diameter, d_(0.9)_, was studied. As additional results, the following parameters were considered: surface-weighted mean diameter (d_[3,2]_) (Equation (2)), volume-weighted mean diameter (d_[4,3]_) (Equation (3)), and Span, or PDI (Equation (4)) [24]. All results were obtained by using the software of the Mastersizer (Mastersizer 2000, version 5.60).

(2) $$d_{\left[3,2\right]}=\frac{\sum n_{i}d_{i}^{3}}{\sum n_{i}d_{i}^{2}}$$

(3) $$d_{\left[4,3\right]}=\frac{\sum n_{i}d_{i}^{4}}{\sum n_{i}d_{i}^{3}}$$

where n_i_ was the number of droplets between two consecutive diameters and d_i_ corresponded to the droplet diameter in µm.

(4) $$\mathrm{PDI}=\frac{d_{\left(0.9\right)}-d_{\left(0.1\right)}}{d_{\left(0.5\right)}}$$

where d_(0.1)_, d_(0.5)_, and d_(0.9)_ were the equivalent volume diameters in µm, at cumulative volumes of 10%, 50%, and 90%, respectively.

### 2.9. Particle Morphology

Three formulations were chosen by their results to evaluate their morphology. Pictures were taken by means of a ESEM Quanta 200 FEI, XTE 325/D8395 (FEI; Hillsboro, OR, USA).

### 2.10. Eugenol Encapsulation Efficiency

Encapsulation efficiency (EE) was calculated from the relation between the experimental and theoretical content of EUG in the polymeric microparticles, thanks to Equation (5):

(5) $$\mathrm{EE}\;(\%)=\frac{\mathrm{Experimental}\;\mathrm{EUG}\;\mathrm{content}}{\mathrm{Theore}\mathrm{tic}\mathrm{al}\;\mathrm{EUG}\;\mathrm{content}}\;\times \;100$$

where theoretical EUG content corresponded to 50.00% in samples whose EUG:polymers ratio was 1:1 and 33.33% in samples whose EUG:polymers ratio was 1:2.

Quantification of EUG was performed by gas chromatography-flame ionisation detector (GC-FID) analysis using azulene as an internal standard. A total of 0.2 g of the microparticles were dissolved in Milli-Q^®^ water, vortexed for 1 min, and sonicated in a bath for 10 min. Then, hexane was added to the solution, which was subsequently vortexed for another minute and centrifuged at 2727 G and 20 °C for 3 min in an Eppendorf 5702 R centrifuge (Hamburg, Germany). The supernatant was transferred to an amber volumetric flask. To ensure the extraction of all EUG, the process was repeated three times. Afterward, the EUG solution was diluted. A total of 10 mL from the diluted solution, along with 5 mL of internal standard solution (3 mg/mL solution of azulene in hexane), was added in a new volumetric flask. Hexane was added to the calibration mark, and samples were prepared in amber vials.

GC-FID analysis was performed with a GC-2010 Plus (Shimadzu, Kyoto, Japan) using an Equity^™^-5 fused silica capillary column (30 m × 0.25 mm, 0.25 µm film thickness) with 5% diphenyl and 95% dimethyl polysiloxane as stationary phase (28089, Supelco^®^, Bellefonte, PA, USA). The following analytical conditions were used: carrier gas helium, flow rate 1.4 mL/min; injection port 250 °C; oven temperature programmed from 100 to 295 °C; and detector at 300 °C.

**Figure 2:**
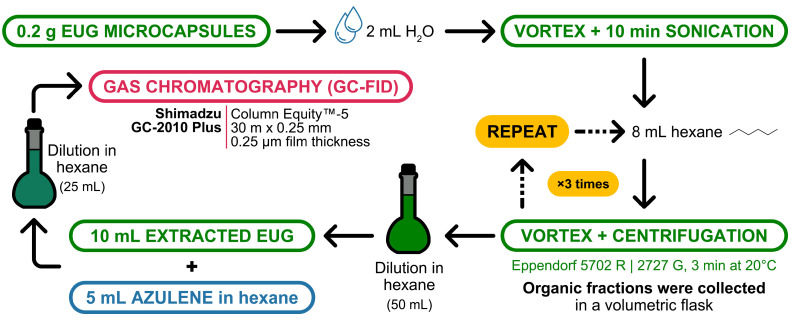
Procedure to extract EUG from the microparticles for GC-FID analysis. Icons retrieved from The Noun Project (CC BY 3.0); credits to Adnan Thariq for the water drops, Sumana Chamrunworakiat for the volumetric flask, and Evgenii Likhachov and Martins Ratkus for the arrows.

Figure 2 shows in detail the EUG extraction procedure to perform the GC-FID analysis.

For a better characterisation of the formulations, this analysis was also performed without dissolving the microcapsules in Milli-Q^®^ water to estimate the amount of EUG that remained on their surface.

### 2.11. Statistical Analysis

The software Statgraphics^®^ Centurion 18 was used to analyse all obtained results.

## 3. Results

The best overall formulations were chosen considering four outcomes: yield of the spray drying process, EUG EE, particle size, and outlet temperature. As the order of Table 2 indicates, the prioritised outcomes were high yield and EUG EE, low particle size, and outlet temperature close to the inlet temperature. Results from all 32 formulations are shown in Table A2 from Appendix A. Additional results are exposed in Table A3 from Appendix A.

### 3.1. Characterisation of Emulsions

**Figure 3:**
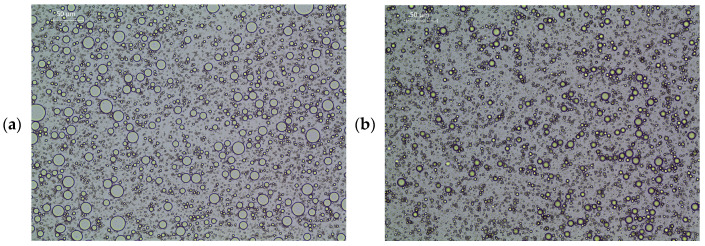
Emulsions before the spray drying process, by optic microscopy at 20x. Both formulations (**a**,**b**) were prepared by high-shear homogenisation at 15,000 rpm. EUG:polymers ratio was 1:1 in (**a**) and 1:2 in (**b**). Images taken by Leica DM 1000 LED optic microscope.

Formulations were oil-in-water (O/W) emulsions before being spray-dried. When analysing them by optical microscopy at 20x (Figure 3), droplets showed wide-size dispersion in both formulations, being higher in Figure 3a. Smaller and less polydisperse droplets corresponded to an EUG:polymers 1:2 ratio (Figure 3b) instead of a 1:1 ratio (Figure 3a).

### 3.2. Outlet Temperature

A closer value to the inlet temperature (110 °C or 130 °C) would provide better microcapsules in terms of smaller particle size and better yield. Of 32 formulations, 20 showed an outlet temperature higher than 60 °C. The best overall three formulations were spray-dried, obtaining outlet temperatures ranging from 55 °C to 78 °C.

### 3.3. Product Recovery

The yield of all 32 assays ranged between 28.80% and 65.35%. A total of 14 formulations had more than 50% of product recovery, remarking the importance that the chosen materials and process variables have in the final product. Product recovery of the best overall three formulations surpassed 57%.

When comparing formulations with different EUG:polymers ratio, the yield was higher as the concentration of MD and GA increased (*p*-value ≤ 0.001).

### 3.4. Particle Size Distribution and Morphology

Particle size, measured as d_(0.9)_, showed different values when studying all 32 formulations. This parameter ranged from 19.60 µm to 164.52 µm, but the 50% of microcapsules were smaller than 48.50 µm.

When comparing the use of Ultra-Turrax^®^ and the paddle stirrer, there were no differences in d_(0.9)_. However, higher flow rate and lower inlet temperature had a greater influence on improving particle size, creating smaller EUG microparticles.

d_(0.9)_ was measured by means of laser diffraction equipment as a basic characterisation parameter of the microparticles. One of the goals in the improvement of the formulations was achieving a high EUG EE in the smaller possible volume, always with the goal of achieving the highest possible product recovery. Therefore, d_(0.9)_ was useful to determine if a formulation had a higher EUG EE due to its larger size or for the effect of any of the five variables from the design of the experiment.

Observed d_(0.9)_ values are usual in spray drying because, among the disadvantages of this technique, it is remarkable for its lack of full control of droplet size and the shape of the produced microparticles [15].

PDI was also analysed as an additional parameter, obtaining high values overall. There were 12 formulations whose PDI ranged between 2.01 and 2.80, but the rest were higher than 3.60. Microcapsules with a lower value of PDI were prepared by using Ultra-Turrax^®^ at 15,000 rpm, although formulations emulsified by means of the paddle stirrer at 390 rpm showed no statistical differences in their PDI. Other research groups that worked with related coating agents and oil extracts obtained similar PDI values when preparing microcapsules by spray drying [13,25].

This parameter was not included in the four studied outcomes to improve microparticles because it is not as relevant as the chosen ones to optimise the coating process. PDI only remarks if the sample has a wide or narrow particle size dispersion. Thus, PDI was not taken into account in the present study, but it was analysed to provide more information about the formulations.

Surface-weighted mean diameter (d_[3,2]_) and volume-weighted mean diameter (d_[4,3]_) in all formulations are also shown in Table A3 from Appendix A, along with d_(0.9)_ and PDI.

Particle morphology of three formulations was also studied by means of a scanning electronic microscope (SEM). Formulations DF06, DF16, and DF20 were chosen due to providing the best result in yield, EUG EE, and d_(0.9)_, respectively (Table 3).

**Figure 4:**
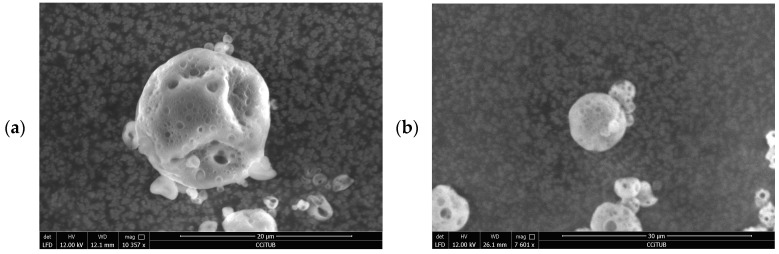

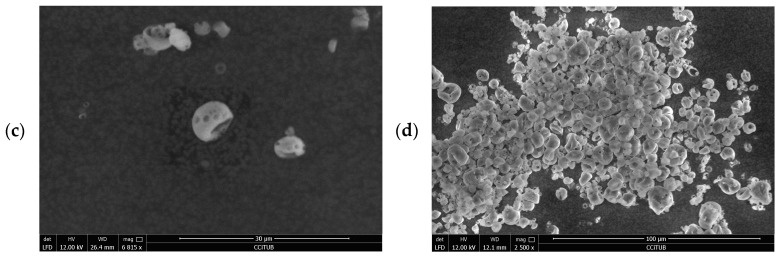
Close view of DF06, DF16, and DF20 ((**a**–**c**) respectively). General view of DF06 (**d**). Images taken by ESEM Quanta 200 FEI, XTE 325/D8395.

As it can be seen in Figure 4, microcapsules had a non-smooth spherical shape with a diameter of less than 20 µm. Figure 4d shows the particle size distribution of DF06. In Figure 4a, DF06 porous spherical morphology, with a diameter of 15.73 µm, can be appreciated. DF16 and DF20 (Figure 4b and Figure 4c, respectively) showed the same characteristics as DF06 but exhibited lower particle size. The diameters of DF16 and DF20 were 9.09 µm and 7.44 µm, respectively.

### 3.5. Eugenol Encapsulation Efficiency

Entrapped EUG was quantified by GC-FID, after dissolving the microcapsules in Milli-Q^®^ water and extracting EUG with hexane. The amount of EUG that remained outside the microparticles was also analysed by using the same technique, without previously dissolving the microcapsules in Milli-Q^®^ water. In this case, results showed that around 1.5% of EUG remained on the surface of the microparticles.

When analysing EUG EE of all 32 formulations, this parameter ranged from 20.82% to 96.07%, although the best three formulations that were selected considering all outcomes, showed an EUG EE between 47.37% and 65.69%.

The main parameter controlling EUG EE was flow rate, increasing the entrapped phenylpropanoid in the microparticles when the emulsion was pumped at 4.5 mL/min (*p*-value ≤ 0.001). Moreover, EUG EE was notably influenced by the combination of flow rate and EUG:polymers ratio (*p*-value ≤ 0.001).

## 4. Discussion

A 2^5^ full factorial design was carried out to optimise the encapsulation of EUG via spray drying. This approach, which included 32 formulations, was chosen to obtain a greater comprehension of EUG coating by MD, GA, and LD to find an optimum workspace and to understand how the whole process could be improved and optimised. By doing so, the effect of five variables affecting the manufacturing process was thoroughly studied. Parameters and outcomes were chosen based on previous knowledge about the technique, as well as bibliographic search. Some remarkable research papers, such as the work of Teodoro et al. [25], were taken into account, along with specific book chapters about the topic and review papers [3,14].

In order to choose the best three formulations, as stated before, it was first prioritised high yield of the spray drying process, secondly a high EUG EE, and, finally, low particle size and high outlet temperature. High product recovery and EUG EE were important to provide a good product by means of an optimum manufacturing process. Small particle size was preferred to achieve a high EUG EE in the minimum possible volume. Hence, d_(0.9)_ was a useful outcome to determine if EUG EE of a formulation was higher than others because the microparticle was bigger or by an improved manufacturing process. A high outlet temperature, as close as possible to the inlet temperature, was the least critical outcome.

Results of the best overall three formulations and their preparation methodology are shown in Table 4 and Table 5, respectively.

It is remarkable that all the best formulations were prepared with a higher ratio of polymers than EUG (1:2, % *v*/*v*), homogenised by Ultra-Turrax^®^, and pumped to the spray dryer at 35 m^3^/h. Yield of the three chosen formulations was around 60%, EUG EE was higher than 47%, and particle size ranged between 22 µm and 55 µm.

The five parameters that were chosen to optimise EUG encapsulation can be divided into the variables affecting the preparation of emulsions or factors affecting the spray drying process.

**Figure 5:**
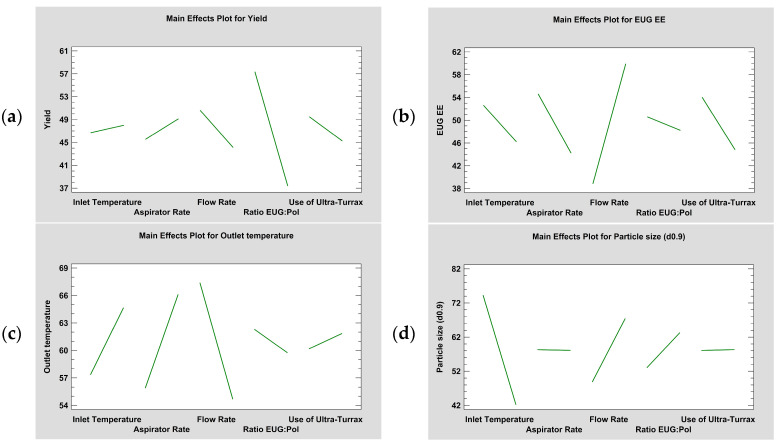
Plots of main effects, by means of Statgraphics^®^ Centurion 18. Yield, EUG EE, outlet temperature, and particle size, as d_(0.9)_, are represented in (**a**–**d**), respectively.

Parameters affecting the formation of O/W emulsions were EUG:polymers ratio (1:2 or 1:1) and the method of homogenisation via Ultra-Turrax^®^ at 15,000 rpm or by paddles at 390 rpm. Both variables mainly affected the yield of the spray drying process (*p* ≤ 0.001) and EUG EE (*p* ≤ 0.001), as can be seen in Figure 5a and Figure 5b, respectively. A 1:2 EUG:polymers ratio improved all outcomes, being the yield of the spray drying process the most influenced. This result, achieving a better yield as the ratio of coating agent increased, has also been observed by other researchers [26,27,28]. The use of Ultra-Turrax^®^ improved both EUG EE and yield (*p* ≤ 0.05 and *p* ≤ 0.001, respectively), without significantly modifying particle size (*p* > 0.05).

Conversely, parameters affecting the spray drying process were flow rate, aspirator rate, and inlet temperature. These variables mostly affected EUG EE, outlet temperature, and particle size, as can be seen in Figure 5b, Figure 5c, and Figure 5d, respectively.

High flow rate (4.5 mL/min) significantly improved EUG EE (*p* ≤ 0.001) (Figure 5b). Low flow rate (1.5 mL/min) improved yield (*p* ≤ 0.001) (Figure 5a), outlet temperature (*p* ≤ 0.001) (Figure 5c), and particle size (*p* ≤ 0.05) (Figure 5d). As there are fewer emulsion droplets in the drying chamber when it is pumped slower, the value of outlet temperature increases, granting better drying of all microparticles.

Aspirator rate, when performing at 35 m^3^/h, improved outlet temperature (*p* ≤ 0.001) and slightly yield (*p* ≤ 0.01), despite at 28 m^3^/h, it managed to increase EUG EE (*p* ≤ 0.05). These results agreed with other research groups [26,29], who also found that yield increased when the aspirator rate was higher. This effect could be the result of reducing the moisture in the drying chamber, favouring the movement of the microparticles to the cyclone and avoiding their retention in the drying chamber.

High inlet temperature (130 °C) reduced the size of the microparticles (*p* ≤ 0.01) and slightly improved yield and outlet temperature (*p* > 0.05 and *p* ≤ 0.001, respectively). Nonetheless, at 110 °C, EUG EE improved, but *p*-value was above 0.5.

**Figure 6:**
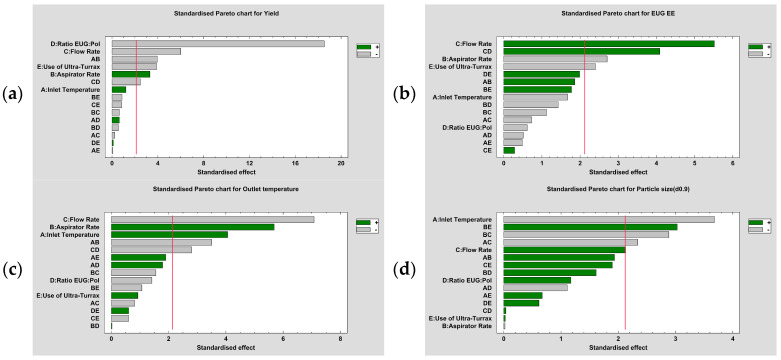
Standardised Pareto analysis, by means of Statgraphics^®^ Centurion 18. Yield, EUG EE, outlet temperature, and particle size, as d_(0.9)_, are represented in (**a**–**d**) respectively.

Studying the combination of factors that improve different outcomes has a great interest in the optimisation of the whole process. This can be achieved by means of a standardised Pareto analysis (Figure 6).

A combination of high inlet temperature (130 °C) and high aspirator rate (35 m^3^/h) improved yield significantly (*p* ≤ 0.001), as can be seen in Figure 6a. Other research groups achieved similar results when using MD as a coating agent [28,29,30]. This combination provided faster microparticle drying and an increase in their collection to the cyclone.

EUG EE improved significantly (*p* ≤ 0.001) if EUG:polymers ratio and flow rate were both in their superior value (1:1 and 4.5 mL/min, respectively), as Figure 6b shows. This combination did not significantly modify particle size (*p* > 0.05).

When studying the standardised Pareto chart for d_(0.9)_, data presented in Figure 6d, it was affected by different combinations of parameters. If inlet temperature or aspirator rate were high in combination to a 4.5 mL/min flow rate, particle size decreased. The emulsification method, in combination with aspirator rate, negatively influenced in d_(0.9)_ (*p* ≤ 0.01).

The main combination of parameters that best improved the outlet temperature (Figure 6c) was the one made up of a high inlet temperature and aspirator rate (*p* ≤ 0.01).

As shown in the results and discussion, analysis of variance was also performed. All *p*-values of the five chosen variables and five combinations of variables are summarised in Table 6.

The main effects plots, as well as the standardised Pareto charts along with the analysis of variance, remark on the importance that flow rate and aspirator rate have on the outcomes. However, the effect that certain combinations of variables have on the whole manufacturing process is particularly interesting. Flow rate and the ratio of polymers in the emulsion are the main variables that, in combination, significantly improved EUG EE. On the other hand, inlet temperature and aspirator rate were the main variables affecting the yield of the spray drying process.

**Figure 7:**
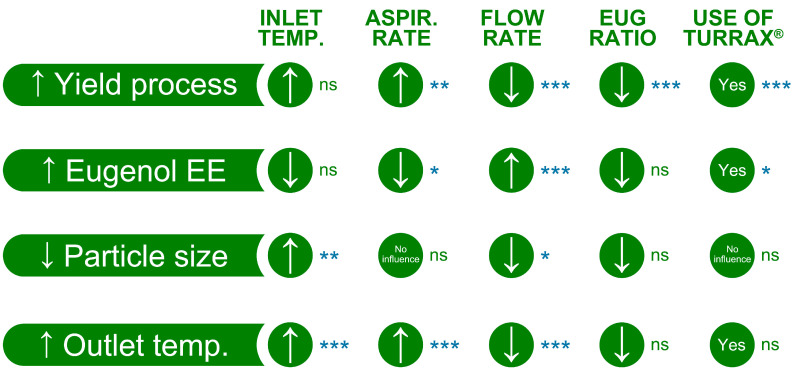
Summary of all results and their statistical significance. An upward-pointing arrow means it is better a high parameter value; a downward-pointing arrow indicates it is better a low parameter value. *p*-values: *p* > 0.05 (ns), *p* ≤ 0.05 (*), *p* ≤ 0.01 (**), *p* ≤ 0.001 (***). *p*-values adjusted at three decimals.

Figure 7 is a summary of all results with their *p*-values, where an upward-pointing arrow indicates that better microparticles were produced when this parameter was in its high value. For instance, to increase the yield of the spray drying process, it is recommended to work with a high aspirator rate, along with preparing the emulsion via Ultra-Turrax^®^. The emulsion, prepared by using a 1:2 EUG:polymers ratio (% *v*/*v*), should be pumped slowly into the spray dryer. In this example, inlet temperature does not significantly affect the yield of the spray dying process, but better results were observed with a high temperature.

When studying the benefits of coating EUG by polymers as MD or cyclodextrins, other research groups proved that the degradation due to light and the oxidation of EUG was minimised [29,30,31]. This protection can be explained by MD film-forming capacities, in addition to the extra shielding that GA confers as a thickener agent [14].

Regarding the obtained yield and EE values in the best formulation (57.22% and 65.69%, respectively), other research groups had similar results [31,32,33]. Encapsulating sour cherry seed oil with MD and GA, Başyiğit et al. achieved a yield of 53.43% and EE of 90.1% in the best formulation [34]. Pino et al. reached higher but similar yield and EE values (63.2% and 77.4%, respectively) when encapsulating winter squash seed oil [28].

This research paper has some strengths, as well as the selection of polymers, to encapsulate EUG, a compound with multiple applications in the alimentary and pharmaceutical fields. All polymers are GRAS-certified by the FDA, achieving more biocompatible and biodegradable formulations, reducing the impact they have on the environment. Furthermore, spray drying is a broadly used technique in research laboratories and industry due to its economic cost and easiness to be used in continuous operation.

Regarding the design of the experiment, as a 2^5^ full factorial design was carried out, the effect of five relevant variables in the spray drying process was thoroughly studied. Usually, researchers study two or three variables when optimising the encapsulation of an oil by spray drying [28,29,33,34,35]. By analysing more factors, the knowledge about the process increases. Thanks to this extensive analysis, it is easier to know which ones are the most relevant variables and how to improve the formulations.

In contrast, the weaknesses of this study could be the use of a pure compound to be encapsulated instead of an EO. EOs are complex products, so it would be interesting to observe if any of their components affect the polymeric coating. Changing the encapsulated product or the coating agents by others GRAS-certified is planned in future studies.

## 5. Conclusions

A 32-experiment factorial design was carried out to understand the process of encapsulation of EUG via spray drying by using natural and biocompatible polymers. The effect of five variables at two levels was studied by means of statistic software, through the analysis of four outcomes from the obtained EUG microparticles.

Overall, the best three formulations had a yield of the spray drying process higher than 57.20%, particle size ranging from 22.40 µm to 55.60 µm, and PDI lower than 2.30. Outlet temperature ranged between 55 °C and 78 °C, and EUG EE was between 47.37% and 65.69%. These results were similar to other research groups that encapsulated vegetal oils by spray drying and using natural polymers, as reflected in the discussion.

Therefore, after statistical analysis of obtained data, the best conditions to prepare EUG microcapsules were high inlet temperature, aspirator rate, and flow rate (130 °C, 35 m^3^/h, and 4.5 mL/min, respectively) and emulsifying via Ultra-Turrax^®^ at 15,000 rpm instead of using a paddle stirrer at 390 rpm. Focusing on the composition of the emulsion, a mixture with fewer amount of EUG than polymers (1:2 EUG:polymers ratio) showed better results.

High aspirator rate and high inlet temperature provided an increase in yield due to a reduction of the moisture in the spray dryer chamber, providing better drying of the microparticles and a faster collection to the cyclone. Flow rate controlled nearly all outcomes, being a high feed velocity, the one that provided better overall microcapsules. By using a high flow rate, the time to produce the microparticles also improved. Using the Ultra-Turrax^®^ and fewer amount of EUG than polymers is recommended because, by doing so, yield increased significantly.

Altogether, the preparation of the emulsion and the whole spray drying process was optimised by means of a 2^5^ factorial design, preparing biodegradable polymeric microcapsules containing EUG.

To take advantage of all obtained results, it would be interesting to encapsulate different EOs instead of a pure compound, using the optimal conditions from this research to study if the same methodology provides similar results. Moreover, MD can be changed to cyclodextrin to compare if there are improvements in the formulations.

## Figures and Tables

**Table 1:** Parameters Involved in the Randomised Factorial Design.

Parameter and Unit	Inferior Value	Superior Value
Inlet temperature (°C)	110	130
Aspirator rate (m^3^/h)	28	35
Flow rate (mL/min)	1.5	4.5
EUG:polymers ratio (% *v*/*v*)	1:2	1:1
Homogenisation via Ultra-Turrax^®^	Yes	No

**Table 2:** Studied Outcomes in the Randomised Factorial Design.

Outcome and Unit	Aim
Product recovery (yield), in %	Maximise
Encapsulation efficiency, in %	Maximise
Particle size [d_(0.9)_], in µm	Minimise
Outlet temperature, in °C	Maximise

**Table 3:** Studied Formulations in a Scanning Electronic Microscope (SEM) and Their Results.

Formulation	Yield (%)	EUG EE (%)	d_(0.9)_ (µm)	PDI	Outlet Temperature (°C)
DF06	65.35	47.37	25.73	2.01	71
DF16	33.72	96.07	164.52	4.55	38
DF20	56.15	45.60	19.60	2.17	67

**Table 4:** Results of the Best Three Formulations, According to Their Overall Performance.

Form.	Yield (%)	EUG EE (%)	d_(0.9)_ (µm)	PDI	Outlet Temperature (°C)
DF22	57.22	65.69	22.41	2.23	55
DF12	63.99	59.15	55.56	2.27	78
DF06	65.35	47.37	25.73	2.01	71

**Table 5:** Conditions to Prepare the Best Three Formulations, According to Their Overall Performance.

Form.	Inlet Temperature (°C)	Aspirator Rate (m^3^/h)	Flow Rate (mL/min)	EUG:Polymers Ratio (% *v*/*v*)	Homogenisation by Ultra-Turrax^®^
DF22	130	35	4.5	1:2	Yes
DF12	110	35	1.5	1:2	Yes
DF06	110	35	4.5	1:2	Yes

**Table 6:** Analysis of Variance (*p*-values) of All Variables, by Means of Statgraphics^®^ Centurion 18. *p* > 0.05 (ns), *p* ≤ 0.05 (*), *p* ≤ 0.01 (**), *p* ≤ 0.001 (***). *p*-values adjusted at three decimals.

Variables and Outputs	Yield (%)	EUG EE (%)	d_(0.9)_ (µm)	Outlet Temperature (°C)
(A) Inlet temperature	0.244 (ns)	0.114 (ns)	0.002 (**)	0.001 (***)
(B) Aspirator rate	0.004 (**)	0.016 (*)	0.984 (ns)	0.001 (***)
(C) Flow rate	0.001 (***)	0.001 (***)	0.049 (*)	0.001 (***)
(D) Ratio EUG:polymers	0.001 (***)	0.547 (ns)	0.259 (ns)	0.178 (ns)
(E) Use of Ultra-Turrax^®^	0.001 (***)	0.029 (*)	0.980 (ns)	0.369 (ns)
AB	0.001 (***)	0.082 (ns)	0.071 (ns)	0.003 (**)
AC	0.813 (ns)	0.475 (ns)	0.033 (*)	0.429 (ns)
BC	0.529 (ns)	0.278 (ns)	0.011 (*)	0.141 (ns)
BE	0.395 (ns)	0.095 (ns)	0.008 (**)	0.303 (ns)
CD	0.024 (*)	0.001 (***)	0.970 (ns)	0.013 (*)

## Data Availability

The original contributions presented in the study are included in the article; further inquiries can be directed to the corresponding author.

## Appendix A

Process conditions to prepare all 32 formulations from the factorial design, and their results, are shown in Table A1 and Table A2, respectively.

**Table A1:** Process Conditions for All 32 Formulations.

Form.	Inlet Temperature (°C)	Aspirator Rate (m^3^/h)	Flow Rate (mL/min)	EUG:Polymers Ratio (% *v*/*v*)	Homogenisation by Ultra-Turrax^®^
DF01	110	28	4.5	1:2	No
DF02	110	35	4.5	1:1	No
DF03	130	35	1.5	1:1	No
DF04	130	28	4.5	1:1	No
DF05	130	35	1.5	1:2	Yes
DF06	110	35	4.5	1:2	Yes
DF07	130	28	4.5	1:2	Yes
DF08	130	28	1.5	1:2	No
DF09	110	35	4.5	1:1	Yes
DF10	130	35	4.5	1:2	No
DF11	110	28	4.5	1:1	No
DF12	110	35	1.5	1:2	Yes
DF13	110	28	4.5	1:2	Yes
DF14	110	35	1.5	1:1	Yes
DF15	110	28	1.5	1:2	No
DF16	110	28	4.5	1:1	Yes
DF17	130	35	4.5	1:1	No
DF18	110	35	4.5	1:2	No
DF19	130	28	1.5	1:1	No
DF20	130	28	4.5	1:2	No
DF21	110	28	1.5	1:1	No
DF22	130	35	4.5	1:2	Yes
DF23	130	35	1.5	1:1	Yes
DF24	110	35	1.5	1:1	No
DF25	130	28	1.5	1:1	Yes
DF26	110	28	1.5	1:1	Yes
DF27	130	28	1.5	1:2	Yes
DF28	130	28	4.5	1:1	Yes
DF29	110	35	1.5	1:2	No
DF30	110	28	1.5	1:2	Yes
DF31	130	35	1.5	1:2	No
DF32	130	35	4.5	1:1	Yes

**Table A2:** Results of All 32 Formulations.

Form.	Yield (%)	EUG EE (%)	d_(0.9)_ (µm)	PDI	Outlet Temperature (°C)
DF01	45.88	42.71	99.72	7.68	51
DF02	29.98	61.33	104.29	7.41	50
DF03	41.41	20.82	52.74	6.09	87
DF04	30.92	46.16	39.57	3.61	58
DF05	59.22	48.51	25.11	2.80	66
DF06	65.35	47.37	25.73	2.01	71
DF07	59.13	54.96	63.76	7.40	64
DF08	56.95	37.78	21.87	2.64	67
DF09	35.42	44.10	34.49	3.81	56
DF10	51.76	38.47	58.10	6.28	60
DF11	30.57	92.95	112.23	5.04	43
DF12	63.99	59.15	55.56	2.27	78
DF13	49.37	82.10	119.85	10.88	45
DF14	44.63	23.44	70.81	9.01	70
DF15	52.87	47.17	23.99	2.60	52
DF16	33.72	96.07	164.52	4.55	38
DF17	28.80	70.36	84.62	4.96	56
DF18	58.49	49.54	88.38	8.89	60
DF19	42.31	28.66	22.77	2.22	72
DF20	56.15	45.60	19.60	2.17	67
DF21	35.87	31.73	22.02	2.04	57
DF22	57.22	65.69	22.41	2.23	55
DF23	46.82	31.10	78.09	8.23	74
DF24	42.93	27.45	90.46	10.31	66
DF25	43.64	33.91	19.86	2.02	67
DF26	34.47	42.06	48.49	5.24	55
DF27	61.93	58.18	61.98	8.19	65
DF28	35.74	77.41	21.48	2.02	47
DF29	60.53	38.63	30.10	4.16	73
DF30	59.56	55.70	70.92	8.25	52
DF31	58.58	37.69	62.53	7.95	76
DF32	37.40	44.11	20.55	2.16	59

Sauter mean diameter (d_[3,2]_), De Brouckere mean diameter (d_[4,3]_), d_(0.9)_ (90% cumulative equivalent volume diameter), and PDI are shown in Table A3.

**Table A3:** Surface-Weighted Mean Diameters (d_[3,2]_) and Volume-Weighted Mean Diameters (d_[4,3]_), d_(0.9)_ and PDI of All 32 Formulations.

Formulation	d_[3,2]_ (µm)	d_[4,3]_ (µm)	d_(0.9)_ (µm)	PDI
DF01	9.20	33.77	99.72	7.68
DF02	9.34	37.39	104.29	7.41
DF03	6.84	20.11	52.74	6.09
DF04	7.94	17.37	39.57	3.61
DF05	6.40	14.03	25.11	2.80
DF06	8.69	14.77	25.73	2.01
DF07	6.63	21.85	63.76	7.40
DF08	6.05	12.43	21.87	2.64
DF09	6.88	17.23	34.49	3.81
DF10	7.11	20.58	58.10	6.28
DF11	13.78	43.12	112.23	5.04
DF12	15.45	30.33	55.56	2.27
DF13	8.22	38.85	119.85	10.88
DF14	6.53	22.46	70.81	9.01
DF15	6.73	14.55	23.99	2.60
DF16	18.64	64.57	164.52	4.55
DF17	11.95	32.15	84.62	4.96
DF18	7.59	28.64	88.38	8.89
DF19	7.34	14.24	22.77	2.22
DF20	6.34	10.91	19.60	2.17
DF21	7.54	13.33	22.02	2.04
DF22	7.11	15.21	22.41	2.23
DF23	7.53	24.33	78.09	8.23
DF24	7.08	27.75	90.46	10.31
DF25	6.94	12.79	19.86	2.02
DF26	7.26	18.32	48.49	5.24
DF27	6.11	19.34	61.98	8.19
DF28	7.37	12.41	21.48	2.02
DF29	5.59	13.17	30.10	4.16
DF30	6.82	22.46	70.92	8.25
DF31	6.27	20.09	62.53	7.95
DF32	6.78	12.75	20.55	2.16
